# Exploiting a Reduced Set of Weighted Average Features to Improve Prediction of DNA-Binding Residues from 3D Structures

**DOI:** 10.1371/journal.pone.0028440

**Published:** 2011-12-08

**Authors:** Yi Xiong, Junfeng Xia, Wen Zhang, Juan Liu

**Affiliations:** 1 School of Computer, Wuhan University, Wuhan, China; 2 Department of Biomedical Informatics, School of Medicine, Vanderbilt University, Nashville, Tennessee, United States of America; University of Georgia, United States of America

## Abstract

Predicting DNA-binding residues from a protein three-dimensional structure is a key task of computational structural proteomics. In the present study, based on machine learning technology, we aim to explore a reduced set of weighted average features for improving prediction of DNA-binding residues on protein surfaces. Via constructing the spatial environment around a DNA-binding residue, a novel weighting factor is first proposed to quantify the distance-dependent contribution of each neighboring residue in determining the location of a binding residue. Then, a weighted average scheme is introduced to represent the surface patch of the considering residue. Finally, the classifier is trained on the reduced set of these weighted average features, consisting of evolutionary profile, interface propensity, betweenness centrality and solvent surface area of side chain. Experimental results on 5-fold cross validation and independent tests indicate that the new feature set are effective to describe DNA-binding residues and our approach has significantly better performance than two previous methods. Furthermore, a brief case study suggests that the weighted average features are powerful for identifying DNA-binding residues and are promising for further study of protein structure-function relationship. The source code and datasets are available upon request.

## Introduction

Protein-DNA interactions play a central role in various biological processes such as gene regulation and transcription [1]. Increasing amounts of structural data on the protein-DNA complexes provide clues to understand the mechanism of protein-DNA recognition, both on the DNA side and on the protein side. Due to the success of structural genomics initiatives, an increasing proportion of solved protein structures are functionally unannotated [2]; however, understanding the relationship between protein structure and function and extrapolating the binding mechanism remains a challenging task. It is well known that a small portion of amino acids on the protein surface are directly involved in protein-DNA interaction. Identification of DNA-binding residues in newly solved protein structures is highly desirable in structural proteomics, which can advance our understanding of the binding mechanism and will be useful in functional annotation and site-directed mutagenesis. In addition, another potential application of DNA-binding residue prediction is in protein-DNA docking, which can be further used to generate models of protein-DNA complexes and study the effects of mutations or different operator sequences on complex formation [3]–[4].

It is relatively straightforward to assign binding residues if the structure of a protein-DNA complex is already known. The binding sites are usually defined in one of the following three ways. The first approach extracts binding sites based on distances between amino acids in a protein and nucleotides in DNA [5]–[22]. The second approach to assign binding residues is based on the difference in the solvent accessible surface area when a protein structure transforms from the non-complexed (the protein without DNA present) to the complexed state (the protein with DNA present) [23]–[25]. Finally, the energy-based methods can be used to define binding sites by calculating the interaction free-energy between atoms in protein and nucleic acid [26]–[27]. Most studies [5]–[22] have defined DNA-binding sites using the first way, in which a cutoff distance (i.e., 3.5 Å∼6 Å) between amino acids and nucleotides are employed to assign DNA-binding sites on proteins.

However, it is a much more complicated task for identifying putative DNA-binding residues on an isolated protein without knowing the structure of its partner (i.e., DNA) or complex. In this case, the experimental procedure is time and resource consuming. This motivates development of high throughput in silico methods for reliable prediction of DNA-binding residues. Over the last decade, two major categories of approaches are well suited to the identification of DNA-binding sites on a protein. The first type is based on the machine learning technique, which attempts to correlate a wide range of features with DNA-binding residues. Although many machine learning-based methods have predicted DNA-binding residues using only protein sequence information [6], [8]–[9], [15]–[16], [18]–[19], [21]–[22], [28]–[29], they are expected to be more accurate when the protein structural information is used [30]. The second type is based on the physical principles that ultimately govern protein-DNA interactions, such as the knowledge-based [12] and docking-based methods [10]. Comparison with the latter type, the machine learning-based methods can be easily and efficiently extended to the inclusion of novel features from a large pool of candidate descriptors, which may be the common properties of DNA-binding residues.

In fact, various properties have been extensively investigated to characterize DNA-binding residues. For example, residues involved in functionally important protein-DNA binding are expected to be more conserved than the rest of the surface [5], [14]; Protein-DNA interfaces have a clear preference for positively charged or polar residues [5], [14], [17], [22]; The positively charged residues have higher solvent accessibility in the interfaces than non interfaces, while for the negatively charged residues the opposite is true [14]; Electrostatic complementarity is also shown to be important for protein-DNA interaction [25], [31]–[35], and DNA-binding sites have a large overlap with the surface patches which show the largest positive electrostatic potential [25]. Our previous study [5] further indicates that the B-factor values vary on DNA-binding residues in DNA-free protein structures, whereas in DNA-bound proteins the average values of B-factor for binding residues are lower than the rest of protein surface, reflecting the fact that DNA-binding regions become rigid upon bound to DNA molecules. Based on the machine learning techniques, some of these features have already been combined for the development of numerous prediction models [5], [14], [17], [20], [22].

In this work, we mainly address three limitations of previous machine learning-based methods. Firstly, the topological features derived from protein residue contact network have provided a novel insight into protein folding, stability and function [36]–[42]. However, the global topological measures of protein structure networks (such as betweenness centrality) have not been used to analyze DNA-binding residues. Secondly, with the increasing number of features used as input for machine learning-based methods, previous studies usually implement feature extraction by directly concatenating descriptors of neighboring residues into a high-dimensional feature vector when using the sliding window strategy on a surface patch. The resultant high-dimensional feature vector increases the possibility of being correlated or redundant among its feature elements. As suggested by Kurgan and co-workers [43]–[44], effective dimensionality reduction can decrease the computational time and complexity of the prediction model, and also provide more insights into the data abundance. Finally, existing studies of protein-DNA interactions treat neighboring residues equally without considering their distance-dependent contributions to the central residues. We believe that this assumption may give distorted information about neighboring residues around DNA-binding residues. Motivated by the aforementioned facts, we use the complex network approach to analyze protein structures and introduce betweenness centrality for the first time to characterize DNA-binding residues, and then implement a dimensionality reduction scheme by extracting the average weighted features on a surface patch, which both results in a reduced feature set and also assigns the distance-dependent weight to each neighboring residue for quantifying its contribution in determining the location of a binding residue.

Based on the above ideas, we propose a novel method (called DBPSite) to identify DNA-binding residues from 3D structure of a protein which does interact with an unknown DNA. A wide variety of experiments have been conducted in the present study. Firstly, we compare the predictive power of weighted average features to that of the concatenated features for representing residues on a surface patch. Next, we analyze the redundancy among the features explored in this work, and then obtain an optimal reduced set of the weighted average features. Using these features, Support Vector Machine (SVM) is employed as the classification engine. Finally, we compare our method DBPSite to two similar methods reported in the recent literatures on the independent tests, which consist of 83 pairs of DNA-binding proteins in holo (DNA-bound) and apo (DNA-free) forms. Experimental results show that DBPSite can predict DNA-binding residues with high accuracy and high efficiency using a reduced set of weighted average features, and compares favorably to two previous methods. A brief case study suggests that the carefully selected weighted average features are indeed powerful for identifying DNA-binding residues and are promising for further study of protein structure-function relationship.

## Methods

### Datasets

We used the same datasets as our previous work [5]. The reason that we used the same dataset is to fairly compare our results with previous studies. The set of 206 nonredundant DNA-binding protein (DBP) chains were divided into two subsets: DBP-123 for training and HOLO-83 for the independent HOLO testing. All chains in HOLO-83 have the structures determined in the absence of DNA. The corresponding unbound structures of HOLO-83 were collected as APO-83 for another independent APO testing.

We focused on identifying DNA binding sites on protein surfaces. For this purpose, a residue is considered as a surface residue if its solvent accessible surface area is at least 10% of maximum value in an extended ALA-X-ALA tri-peptide state [14]. A surface residue is defined to be a binding residue if any of its heavy atoms is within 4.5 Å of any heavy atom in a neighboring DNA molecule [5], [20]. The rest of surface residues are assigned as nonbinding residues. According to the above definition, DBP-123 contains 2903 binding residues and 15420 nonbinding residues, HOLO-83 includes 2024 binding residues and 11818 nonbinding residues, and APO-83 includes 1901 binding residues and 11769 nonbinding residues, respectively.

### Feature set construction

To build a classifier that can discriminate DNA-binding from nonbinding residues, we extracted features based on evolutionary profiles, interface propensity, topological and structural features. For each target (or central) residue, the feature vector is usually constructed by the sliding window on a surface patch (For each surface residue, its distances by their alpha C atoms with all other surface residues in the same protein chain are calculated and sorted in ascending order, and then the *L* spatially nearest surface residues constitute a surface patch) for including the environmental information. In our study, we set *L* = 25 as the optimal size for building the surface patch (see details in Results and Discussion section). Previous methods for predicting DNA-binding residues included data for neighboring residues by concatenating their properties, resulting in high-dimensional feature vectors.

To avoid the large size of feature vectors, we proposed a condensed encoding scheme by a weighted average over the properties of neighboring residues. Our results (see Results and Discussion section) indicated that there are clear advantages in using the weighted average features rather than concatenating properties for neighboring residues.

#### Definition of the weighting factor

The weighting factor, which weights the contribution of each adjacent residue according to its relative distance to the central residue, is represented as follows:
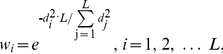
(1)where *w_i_* is the weighting factor for residue *i* on a surface patch with a size of *L*; *d_i_* is the distance between residue *i* and the central residue. If the residue *i* is the central residue itself, the *w_i_* will have a maximum value of 1.

#### Evolutionary profile of the surface patch

The position specific scoring matrix (PSSM) was generated by three iterations of PSI-BLAST [45] searches against NCBI nonredundant database (ftp://ftp.ncbi.nlm.nih.gov/blast/db/), in much the same way as previous work [5], [11]. The PSSM elements were scaled to the range [0, 1] by a standard logistic function [46]. The concatenated PSSM profile (C-PSSM) for a surface patch is constituted by concatenating the vector elements of PSSM for all neighboring residues. Since 20 log-odds values are utilized to represent PSSM profile for a residue, a feature vector with the full size of L*20 would be constructed as the C-PSSM for a surface patch of *L* residues. When the dimension of input data is too large and it is suspected to be redundant, it is desirable to transform the input data into a reduced representation set of features (dimensionality reduction). The solutions to this problem can roughly be grouped into two main categories: feature selection and feature extraction. Feature selection produces a subset of the original features, whereas feature extraction creates new features resulting from the combination of the original features. In this work, we proposed a novel PSSM profile (called RW-PSSM) to reduce the feature dimensionality by combining intuitive feature selection and feature extraction. The RW-PSSM profile is composed of two parts. The first part is a *1-by-20* vector, corresponding to PSSM profile for the central residue. The second part is also a *1-by-20* vector, one element corresponding to a weighted average value over the neighboring residues for one amino acid type. For *a-th* amino acid type, the entry *F_a_* is generated by weighted averaging over the *a-th* column of PSSM for all residues on a surface patch. The entry *F_a_* is given by:
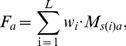
(2)where

1. *i* is the index of a residue on a surface patch; *s(i)* is the sequential index for residue *i*;
2. *M_s(i)a_* is the value of the *a*-th type of amino acid for the *s(i)-th* amino acid in the protein sequence.

**Figure 1:**
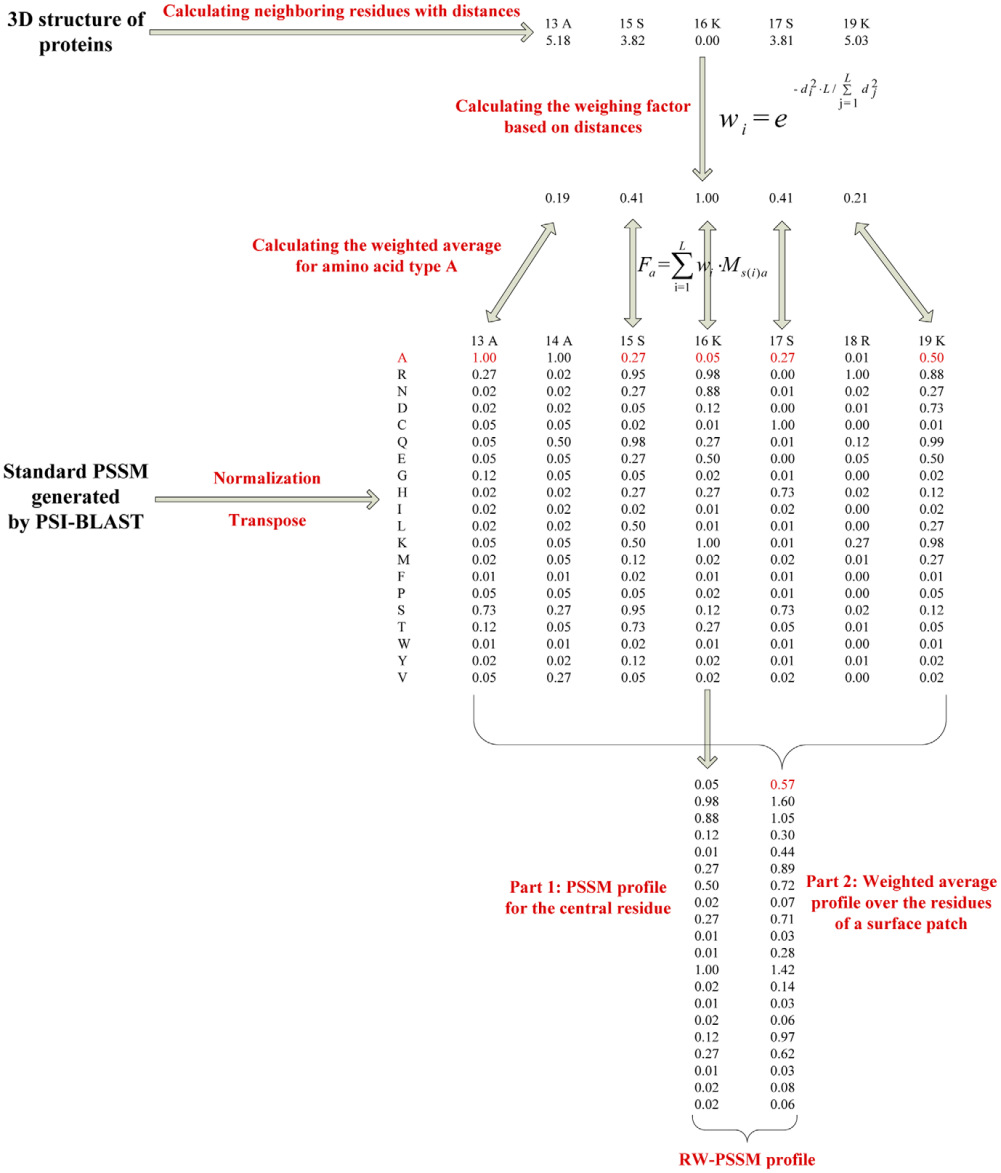
Flowchart of generating the RW-PSSM profile. Given a DNA-binding protein (PDB id: 1A02; Chain: A), a size of 5 for the surface patch is set for simple illustration. The central residue is 16K (Seq id; residue name), with its four neighboring surface residues (i.e., 17S, 15S, 19K, 13A).

As a result, the RW-PSSM feature vector for a residue has a fixed length of 40-dimension, irrespective of the size of a surface patch around the central residue. Fig. 1 shows an example about generating the RW-PSSM profile vector.

#### Betweenness centrality

Protein structures are recast as topological graphs based on protein residue contact maps, where each vertex of the graph represents the alpha C atom of an amino acid and edges connect vertices within a distance cutoff of 8 Å [37], [47]. Once the graph is constructed, a variety of topological metrics can be used to describe functional residues.

Betweenness centrality (BC) measures how frequently a vertex occurs on the shortest path between all other vertex pairs within the contact graph (undirected graph) of a protein chain of length *n*. Since the chains vary in length, the measure is normalized by dividing through the number of pairs of vertices not including *v*, which is *(n−1)(n−2)/2*.
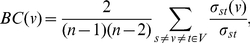
(3)where *V* is the set of vertices, 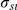 is the number of shortest paths from *s* to *t*, and 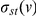 is the number of shortest paths from *s* to *t* that pass through vertex *v*.

The weighted average betweenness centrality for a surface patch was calculated from the betweenness centrality values of component residues, weighted by the weighting factor *w_i_* of residue *i*:
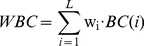
(4)


#### Interface propensity

Interface propensity (*IP*) describes the relative importance of the different types of amino acids in DNA-binding interfaces. The propensity values were calculated using the protein-DNA pairs on the chosen dataset as follows:
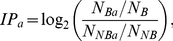
(5)where *N_Ba_* is the number of DNA-binding residues for a particular amino acid type *a*, *N_B_* is the number of all DNA-binding residues, *N_NBa_* is the number of nonbinding residues for a particular amino acid type *a*, and *N_NB_* is the number of all nonbinding residues. In 5-fold cross validation on DBP-123 dataset, the interface propensity is derived iteratively from 4 of the 5 five subsets, and tested on the remaining one subset independently. In independent tests on HOLO-83 and APO-83, the interface propensity is obtained on the whole training set DBP-123.

These propensity values are centered around 0. A positive propensity value indicates that a particular amino acid occurs more frequently in DNA-binding interface than on the surface of the protein. A negative propensity value indicates that an amino acid occurs less frequently in the interface than on the surface of the protein. The weighted average interface propensity was defined as:
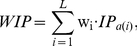
(6)where *a(i)* refers to the amino acid type of residue *i*.

#### Accessible surface area

The program NACCESS [48] was employed to calculate residue accessible surface area (ASA) and relative solvent accessibility (RSA) values. Five pairs of ASA and RSA based attributes were constructed: AaASA and AaRSA (all atoms), McASA and McRSA (all main chain or backbone atoms), ScASA and ScRSA (all side chain atoms, including alpha carbons), ApASA and ApRSA (all polar side chain atoms, i.e., oxygen and nitrogen atoms), and NpASA and NpRSA (all non-polar side chain atoms, i.e., non-oxygen and non-nitrogen atoms). Since most of these attributes are highly correlated or redundant [49], it cannot yield satisfied performance using all of them. We used only ScASA attribute of the central residue on a surface patch. The weighted average ScASA (WScASA) was defined in the same as other features (such as WBC and WIP).

### Model construction and evaluation

In the present study, SVM classifiers were applied for prediction of DNA-binding sites. SVM models were implemented with the radial basis function as a kernel using the *e1071* library in R (http://cran.r-project.org/web/packages/e1071/), which provides the interface to the LibSVM [50]. The models were evaluated by 5-fold cross validation on DBP-123, in which the 123 protein chains were randomly divided into five subsets (folds). The overall performance was obtained by averaging the performance of the five subsets (at the fold level). Furthermore, our proposed model was validated by the independent tests on the bound and unbound structures (HOLO-83 and APO-83) respectively. On the independent datasets, the final performance is summarized by averaging the performance of 83 protein chains. Since the numbers of DNA-binding and nonbinding residues in proteins are highly unbalanced, the classifiers were trained using all binding residues and an equal number of nonbinding residues chosen randomly from the training set. It is worth mentioning that nonbinding residues are not removed from the testing set in cross validation and independent tests. Removing nonbinding residues will yield a biased measure of prediction performance since the identity of nonbinding residues is unknown beforehand for an actual prediction.

It is a nontrivial task to assess the quality of prediction for heavily unbalanced datasets such as this one. In our training set, ∼15% of the samples belong to one class (binding residues). In the testing set, 38/166 protein chains have the ratios of binding residues between ∼3% and ∼10%. In such cases, the accuracy and Receiver Operator Characteristic curves can present overly optimistic assessments of an algorithm's performance [51]. Thus, we focus on the precision-recall (PR) curve [51], which is a plot of the recall (also called sensitivity) versus precision for a binary classifier at varying thresholds. In addition, we used F-measure (*F_1_*), which is a harmonic mean of recall and precision. These metrics are defined as follows:
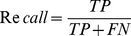
(7)

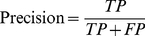
(8)

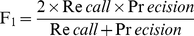
(9)where *TP* is the number of correctly predicted DNA-binding residues, *TN* is the number of correctly predicted nonbinding residues, *FP* is the number of nonbinding residues predicted as binding residues and *FN* is the number of binding residues wrongly predicted as nonbinding.

We also used the area under the PR curve (PR-AUC) as the main metric, which is calculated by the AUCCalculator program [51]. The significance of the difference between two different methods is assessed using the Wilcoxon signed rank test over paired performance statistics for all protein chains in the dataset.

## Results and Discussion

To overcome three limitations of previous machine learning-based methods for DNA-binding sites identification, in our experiments, we firstly validated the solutions for three limitations respectively: the RW-PSSM profile compares favorably to the conventional C-PSSM profile; Several topological and structural features are proved again to have satisfactory ability to describe DNA-binding residues on proteins, especially for the betweenness centrality; And for those highly predictive features, we have carefully rank their importance and combination on the improvement of DNA-binding sites prediction. Then, a brief case is further deeply studied to reflect the predictive power of our proposed weighted average features. Finally, a variety of comparisons between DBPSite and two previous methods strongly support the superior accuracy and efficiency of our method.

### Predictive power of RW-PSSM profiles

**Figure 2:**
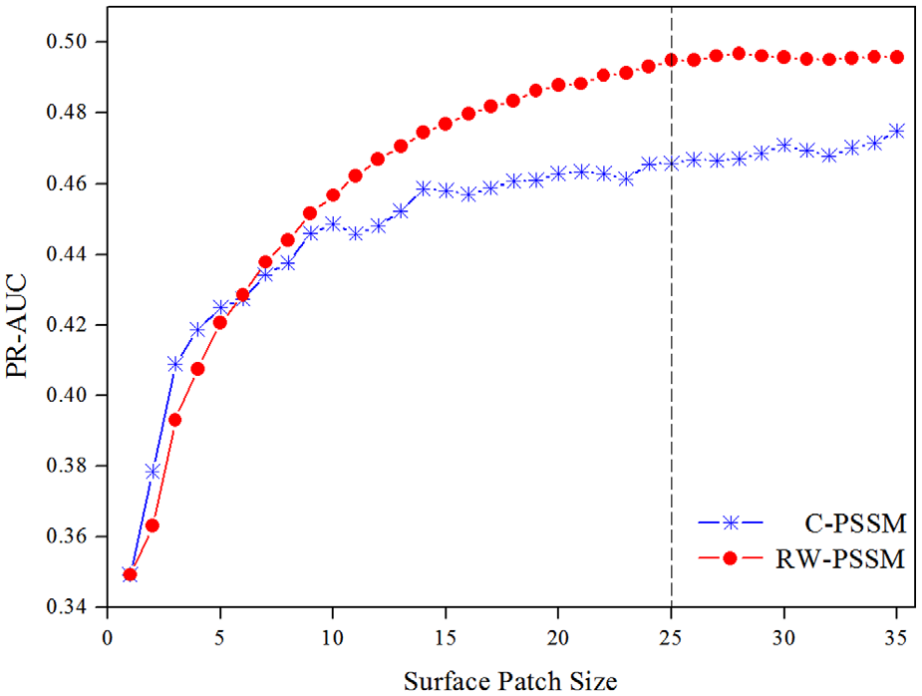
Performance comparison of RW-PSSM and C-PSSM. The comparison was conducted using 5-fold cross validation on DBP-123 dataset at surface patches of varying sizes.

In this section, we compared performance of RW-PSSM with C-PSSM profiles in terms of PR-AUC on the training dataset DBP-123 using 5-fold cross validation. Fig. 2 shows the prediction performance (PR-AUC) of SVM classifiers over increasing patch sizes from 1 to 35 for the two PSSM profile encoding schemes. As shown in Fig. 2, if only the feature for the target residue was used as input, the PR-AUC scores were lower for the two types of classifiers. However, as we increased the patch sizes for inclusion of more neighboring surface residues, their performance was remarkably improved. The results suggest that the local environment around the target residue indeed contributes to the prediction of DNA-binding residues.

More importantly, the classifier with RW-PSSM as input consistently performs better than that with C-PSSM as input when the patch sizes are greater than 5. The observation confirms our hypothesis that assigning distance-dependent weights to neighboring residue and weighted averaging is helpful for determining the location of binding residues. A closer examination of Fig. 2 reveals that both profiles reach a plateau for a patch size of roughly 25 residues, with the RW-PSSM achieving a top PR-AUC of 0.495 and C-PSSM obtaining 0.466. The improvement of the overall performance is promising, considering the fact that RW-PSSM used a significantly lower size of 40-dimension in the input vectors than the sizes of 500 (20*25) for C-PSSM. As a result, the RW-PSSM profiles are several orders of magnitude faster to train and test than the conventional C-PSSM profiles. Therefore, the RW-PSSM profiles are adopted to construct the classifiers in our study.

### Analysis of interface propensity, betweenness centrality and side chain accessible surface area

In addition to the RW-PSSM profile, we investigated other features (including interface propensity, betweenness centrality and side chain accessible surface area) that have high predictive power for DNA-binding residues, with the final goal to improve performance by combining highly predictive features with the RW-PSSM profile. As discussed in previous section, prediction performance is usually varied with surface patch sizes when these features are used for weighted averaging over a patch of residues. Theoretically speaking, the optimal patch size for the RW-PSSM profile is not necessarily optimal for other features. However, to make different features for stringent comparison and fewer parameters for tuning, we simply used the same surface patch size of 25 and did not optimize the patch sizes individually for all the features explored. Using 5-fold cross validation on DBP-123, we calculated the predictive power of IP, BC and ScASA when they are individually used with only information of the target residue or weighted information of neighboring residues (see Table 1).

**Table 1:** Predictive power of individual feature on the DBP-123 dataset by 5-fold cross validation.

Feature	Recall (%)	Precision (%)	F_1_	PR-AUC
IP	66.4	22.3	0.332	0.228
WIP	71.9	30.1	0.423	0.326
BC	36.8	21.9	0.274	0.208
WBC	65.1	22.6	0.333	0.228
ScASA	29.4	21.1	0.238	0.203
WScASA	41.7	21.3	0.279	0.211

Table 1 shows clearly that interface propensity performs best among the features of IP, BC and ScASA. The result is not surprising due to the following fact. There exists a clear preference of amino acid types (i.e., positively charged or polar residues) for DNA-binding interfaces, which are in agreement with previous studies [5], [14]. Moreover, as shown in Table 1, the weighted interface propensity performers better than interface propensity of the central residues.

**Figure 3:**
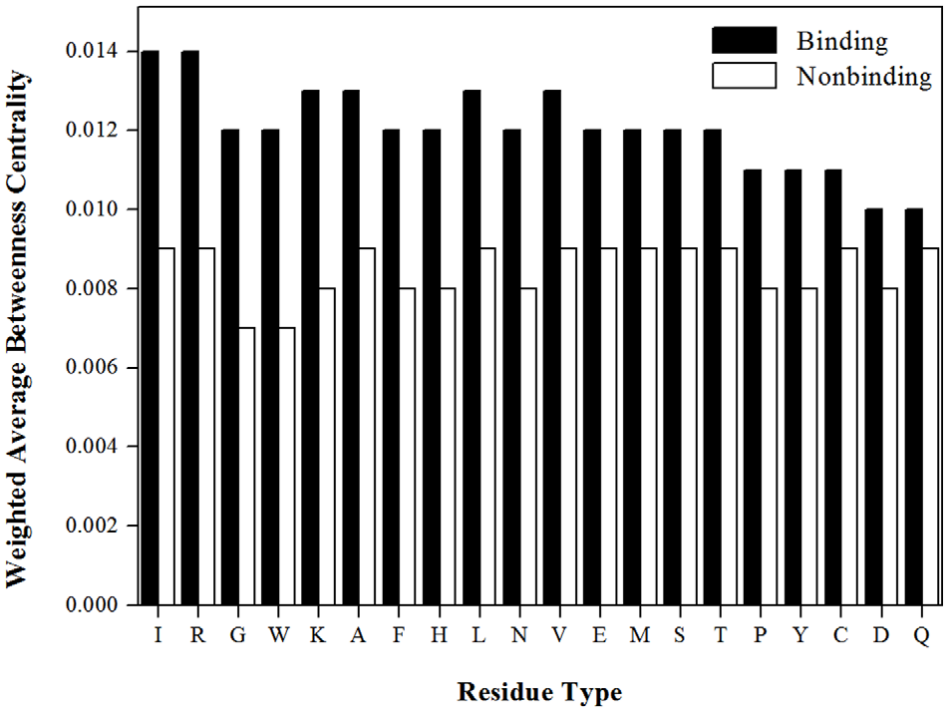
Distribution of the weighted average betweenness centrality. This figure shows the distribution of the 20 types of residues for the weighted average betweenness centrality between DNA-binding and nonbinding residues on DBP-123 dataset. The abscissa is in descending order of the difference of vertical axis between DNA-binding and nonbinding residues.

Previous studies have indicated that betweenness centrality is well correlated with hot spot residues in protein-protein interfaces [42] and RNA-binding residues in protein-RNA interfaces [37]. The present study also found that residues located at protein-DNA interfaces exhibit the central role in the protein network with high betweenness centrality. It is shown in Table 1 that the predictive power of betweenness centrality was low (PR-AUC 0.208) for individual residues but was high (PR-AUC 0.228) when averaged over a patch of neighboring residues. This may suggest that a set of residues with higher betweenness centralities form a community so as to play an important role in protein-DNA interaction. As shown in Fig. 3, DNA-binding residues are distinguishable from the nonbinding residues on protein surfaces by their higher weighted average betweenness centrality.

In our work, we also investigated other centrality measures derived from the protein residue contact map, such as degree centrality and closeness centrality. Since they are highly correlated with betweenness centrality, we retained betweenness centrality with the highest predictive power among the centrality measures (see Table S1).

The property of residue solvent accessibility has been used for the prediction of DNA-binding residues in previous studies [5], [14]. We used accessible surface area of side chains, since the contribution of proteins to protein-DNA interfaces comes mostly from side chains [52]. The results in Table 1 indicate that the predictive power of ScASA for individual residues was comparable to that of the weighted averages over a patch of neighboring residues.

### Evaluation of feature importance and combination of highly predictive features

In this section, the selected features in previous sections were combined to evaluate their performance using 5-fold cross validation on the DBP-123 dataset. Our analysis indicates that the high correlation coefficient for BC and WBC is 0.61 (0.44 for ScASA and WScASA, 0.39 for IP and WIP). It is not a good idea to combine the redundant features as input for classifiers [49], [53]. Therefore, we only retained one feature with higher predictive power from two of them. As to side chain accessible surface area, we used ScASA for the target residue since it has relatively lower correlation coefficient with other features such as WIP and WBC, although WScASA shows slight better performance than ScASA for the target residue when each one is used individually. As a result, we kept a combination of RW-PSSM, WIP, WBC and ScASA as the feature set for the final model for training and testing, which shows the best performance in the 5-fold cross validation (Table 2).

**Table 2:** Prediction performance by leaving out one feature at a time on the DBP-123 dataset by 5-fold cross validation.

Feature	Recall (%)	Precision (%)	F_1_	PR-AUC
All features	80.0	39.2	0.524	0.522
Without RW-PSSM	73.7	31.8	0.443	0.360
Without WIP	78.3	38.1	0.511	0.506
Without ScASA	79.5	38.5	0.518	0.513
Without WBC	79.3	39.1	0.521	0.520

To re-evaluate the feature importance, we measured the prediction performance of the 5-fold cross validation by leaving out one of the features at a time. Table 2 shows that the prediction performance was declined in comparison to that of the classifier using all features when leaving out each feature in describing these residues. For instance, when we removed the RW-PSSM, the PR-AUC score is significantly decreased from 0.522 to 0.360. A closer examination of Table 2 shows that the WBC and ScASA are comparable, but both of which are interior to WIP, and WIP is interior to RW-PSSM. The observation, that the predictive power of WBC and ScASA is comparable when they are combined with RW-PSSM, contradicts the result in previous section that WBC is more powerful than ScASA. This inconsistency can be explained by the fact that WBC is positively correlated (Pearson's correlation coefficient is 0.37) with residue conservation scores generated from multiple sequence alignment and part of the residues with high weighted averaged betweenness centrality are also conserved residues of proteins. This result is consistent with the finding of another study [42] that most of the statistically significant high-betweenness residues, which were conserved in sequence alignments, comprised of most of hot spot residues or residues in contact with hot spots.

### A brief case of weighted average features for structure-function analysis

**Figure 4:**
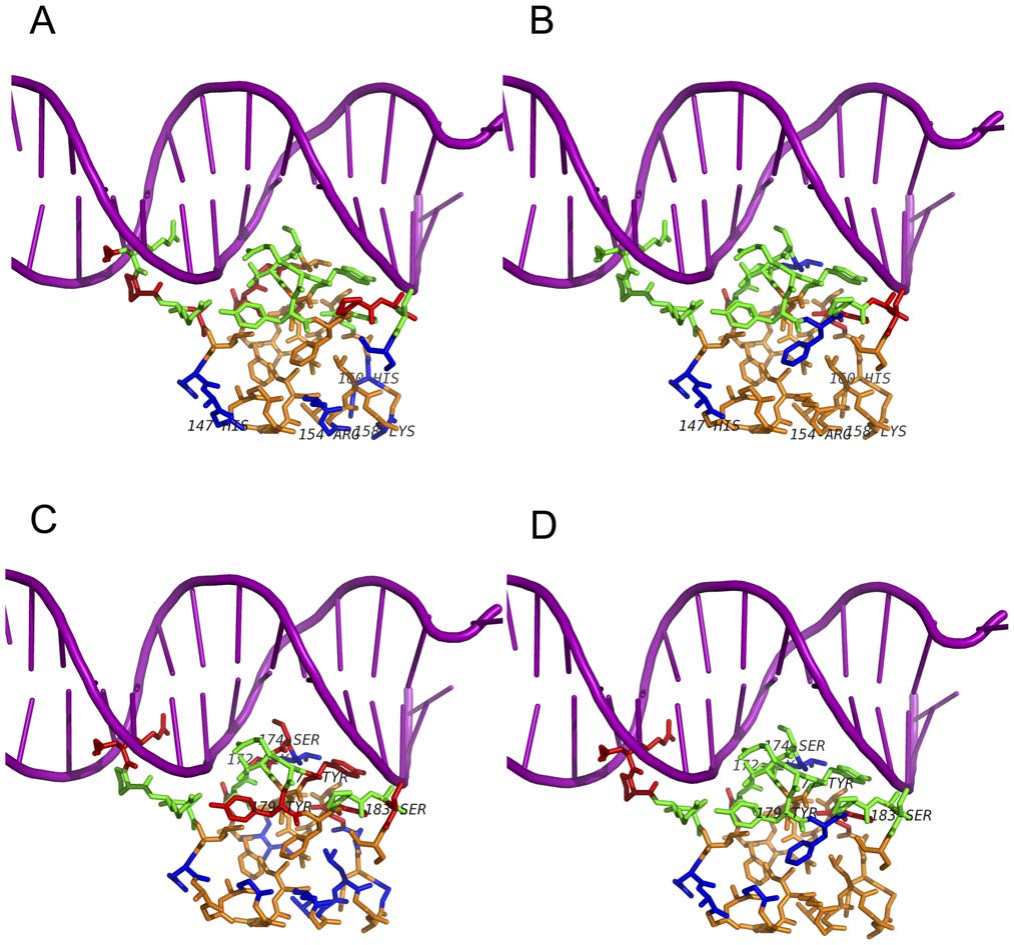
Prediction results shown on 1JKO using four different rank-based methods. (**A**) Interface propensity-based method. (**B**) Weighted average interface propensity-based method. (**C**) Betweenness centrality-based method. (**D**) Weighted average betweenness centrality-based method. Colors of different residues are defined as follows: green denotes true positives (TP), blue denotes false positives (FP), orange denotes true negatives (TN), and red denotes false negatives (FN). Purple cartoon denotes the DNA molecules.

To clearly demonstrate the predictive power of our selected weighted average features, we give an example of the 1JKO (PDB id), which is the Hin recombinase in complex with DNA [54]. The protein has 17 DNA-binding residues (GLY 139, ARG 140, PRO 141, ARG 142, ALA 143, ARG 162, GLY 170, ILE 171, GLY 172, SER 174, THR 175, TYR 177, ARG 178, TYR 179, PRO 181, ALA 182, SER 183). We considered the top-ranked 17 residues as candidate DNA-binding residues. On this protein chain, the interface propensity-based method achieved a recall and precision at 52.9%, whereas the weighted average interface propensity-based method boosted the recall and precision at 76.5%. The interface propensity-based method misclassified some of positively charged residues (such as ARG 154, LYS 158, HIS 147 and HIS 160) as DNA-binding residues (Fig. 4A, B). However, the weighted average interface propensity-based method can rectify the misclassification results, and correctly predicted the four positively charged residues as nonbinding residues.

Similarly, we used other feature (i.e., betweenness centrality) to rank the surface residues, and considered the top-ranked 17 residues as DNA-binding residues. The betweenness centrality-based method obtained a recall and precision at 47.1%, while the weighted average betweenness centrality-based method yield better performance with the recall and precision at 70.6%. The betweenness centrality-based method misclassified the binding residues (such as GLY 172, SER 174, TYR 177, TYR 179 and SER 183) with lower betweenness centrality as nonbinding residues (Fig. 4C, D). However, the five residues have the high weighted average betweenness centrality and they are correctly predicted as binding residues when using the weighted average betweenness centrality-based method.

### Independent tests and comparison with other methods

A true test of any prediction approach is to make predictions for the datasets not utilized in training. In the section, we first evaluated the prediction performance of DBPSite on the independent datasets of HOLO-83 and APO-83. As shown in Table 3, the PR-AUC scores on HOLO-83 and APO-83 (0.550 and 0.543) are even higher than the performance (PR-AUC: 0.522) of cross validation test on the training set. Actually, it is not fair to compare the PR-AUC scores in this case, since the PR-AUC for the training set is calculated on the fold level but the PR-AUC for testing set is derived on the protein chain level. Instead, we conducted the independent tests with the main purpose of comparing DBPSite to previously published methods.

**Table 3:** Prediction performance on the independent test sets of HOLO-83 and APO-83.

Method	Recall (%)	Precision (%)	F_1_	PR-AUC	*P*-value (PR-AUC)
Our previous method [3]	68.9	41.1	0.483	0.499	
DBPSite	73.1	43.1	0.511	0.550	<0.001
Our previous method	70.1	41.1	0.482	0.510	
DBPSite	72.3	43.5	0.500	0.543	<0.01

Recently, many sequence-based methods have been developed to predict DNA-binding residues [6], [8]–[9], [15]–[16], [18]–[19], [21], [28]–[29]. Since they did not use protein structure information, it is unfair to compare our DBPSite predictor with them. Two published methods, including DISPLAR [14] and our previous method [5], have the most resemblance to DBPSite. These methods utilize the spatial PSSM as the main feature and the machine learning techniques to predict DNA-binding residues, given the structure of a protein which is known to interact with DNA. A detailed description of the differences of the three methods is given in Table S2.

The DISPLAR method [14] has two types of input: PSSM profile and solvent accessibility. The prediction for each residue was based on the input variables of the considering residue itself plus 14 of its nearest spatial neighbors, which constituted a long input vector with the size of 315-dimension. DISPLAR uses a different cutoff distance of 5 Å to define a binding residue. For a fair comparison, we retrained our model on the DBP-123 dataset using the same cutoff distance. In the absence of PR-AUC value (the PR-AUC cannot be calculated since the single-threshold value was used for prediction in DISPLAR), the prediction performance of DBPSite were reported at the same recall or precision as that of DISPLAR for comparisons. As shown in Table 4, our method achieved significant better performance than that of DISPLAR on the HOLO-83 dataset, i.e., for the same recall 46.4%, our method had a higher precision of 59.7% compared to that of 51.3% in DISPLAR. In APO-83, our method performed significantly better than DISPLAR. On the same recall of 40.9%, our approach had a considerably higher precision of 56.9% than that of 45.2% obtained by DISPALR.

**Table 4:** Performance comparison of DBPSite with DISPLAR on the test sets of HOLO-83 and APO-83.

Dataset	Method	Recall (%)	Precision (%)	F_1_	*P*-value (F_1_)	*P*-value (Precision)	*P*-value (Recall)
HOLO-83	DISPLAR [12]	46.2	51.3	0.451			
	DBPSite (0.00)	72.0	45.7	0.529	<0.02		
	DBPSite (0.69)	46.3	59.7	0.478		<10^−4^	
	DBPSite (0.25)	64.8	51.3	0.537			<10^−5^
APO-83	DISPLAR	40.5	45.2	0.391			
	DBPSite (0.00)	71.5	47.0	0.523	<10^−5^		
	DBPSite (0.76)	40.9	56.9	0.427		<0.001	
	DBPSite (−0.07)	73.9	45.4	0.523			<10^−13^

Our previous work [5] used four types of input: PSSM profile, solvent accessibility, packing density and pK_a_. The prediction for each residue was based on the input attributes of the considering residue itself and 10 of its nearest spatial surface residues, which constituted a high-dimensional input vector with the size of 253-dimension. The results summarized in Table 3 show that DBPSite had significant higher prediction power in terms of PR-AUC accuracy than our previous method on the HOLO-83 and APO-83 datasets.

Comparison to such two methods, DBPSite used a significantly lower size of 43-dimension in the input vectors, making it faster and more accurate. The observations above clearly demonstrate that the DBPSite method outperforms the two previous methods.

### Conclusions

The main goal of the current study is to provide valuable insights into DNA-binding residues and improve the prediction performance of DNA-binding sites from the unbound structure of a protein which interacts with unknown DNA.

Our study indicates that the betweenness centrality, one of the global topological central measures, can be used to discriminate DNA-binding residues from the remaining surface. The results further demonstrated that the predictive power of betweenness centrality was low for individual residues but was high when weighted averaging over a patch of neighboring residues, suggesting that a set of residues with higher betweenness centralities form a community to play an important role in protein-DNA interaction.

The weighted average representation scheme has been proved to be efficient and effective to this classification task. For example, the traditional C-PSSM profile on a surface patch is a high-dimensional vector in direct proportion to the size of the patch, whereas our proposed RW-PSSM profile has a 40-dimensional feature vector, irrespective of the size of the patch. Experimental results show that the latter are several orders of magnitude faster to train and test than the former, and the RW-PSSM profiles can be favorably combined with other features to boost the performance of predicting DNA-binding residues. The weighted average scheme can potentially be expanded to predict other functional sites, such as protein-protein and protein-RNA interaction residues.

The experiments on independent tests suggest that our method DBPSite significantly (validated by the P-value of Wilcoxon signed rank test) outperforms two similar published methods for prediction of DNA-binding residues from 3D structure of a protein.

## Supporting Information

Table S1 Predictive power of individual centrality measure on the DBP-123 dataset by 5-fold cross validation.
(PDF)

Table S2 Methodological differences between DISPLAR, our previous method and our present method (DBPSite).
(PDF)
