# Supplementary material for: Exploiting a Reduced Set of Weighted Average Features to Improve Prediction of DNA-Binding Residues from 3D Structures
Source: PLoS One. 2011 Dec 8;6(12):e28440. doi: 10.1371/journal.pone.0028440 (PMC3234263; doi:10.1371/journal.pone.0028440)
Supplement: Table S1 — Predictive power of individual centrality measure on the DBP-123 dataset by 5-fold cross validation. (PDF) [file pone.0028440.s001.pdf]

Supporting Information Table S1

In addition to betweenness centrality (*BC*), we analyzed the predictive power of other two types of centrality measures [1]: degree centrality (*DC*) and closeness centrality (*CC*).

Degree centrality

The degree centrality of a vertex is defined as the number of edges that connect the vertex with its neighboring vertices within the contact graph of a protein chain.

Closeness centrality

Closeness centrality is calculated as the reciprocal of the average geodesic distance (i.e., the shortest path) between a vertex *v* and any other vertex *t* within the contact graph *G* of a protein chain of length *n*:

$$CC(v)=\frac{n-1}{\sum_{t\in V\backslash v} d_G(v,t)}$$

Table S1. Predictive power of individual centrality measure on the DBP-123 dataset by 5-fold cross validation.

Feature	Recall (%)	Precision (%)	F_1	PR-AUC
BC	36.8	21.9	0.274	0.208
WBC	65.1	22.6	0.333	**0.228**
CC	58.4	20.1	0.299	0.202
WCC	68.9	19.8	0.307	0.206
DC	50.9	16.8	0.248	0.168
WDC	46.0	18.7	0.255	0.205

References

1. Maetschke SR, Yuan Z (2009) Exploiting structural and topological information to improve prediction of RNA-protein binding sites. BMC Bioinformatics 10: 341.
