# Supplementary material for: Exploiting a Reduced Set of Weighted Average Features to Improve Prediction of DNA-Binding Residues from 3D Structures
Source: PLoS One. 2011 Dec 8;6(12):e28440. doi: 10.1371/journal.pone.0028440 (PMC3234263; doi:10.1371/journal.pone.0028440)
Supplement: Table S2 — Methodological differences between DISPLAR, our previous method and our present method (DBPSite). (PDF) [file pone.0028440.s002.pdf]

Supporting Information Table S2

Table S2. Methodological differences between DISPLAR, our previous method and our present method (DBPSite).

Difference	DISPLAR [1]	Our previous method [2]	Our present method (DBPSite)
**Features analyzed and used**	PSSM, Solvent accessibility	PSSM, Solvent accessibility, Side chain pK_a, Packing density and B-factor	PSSM, Side chain ASA, Propensity and Betweenness centrality
**Distance of neighboring residues**	No	No	Yes
**Global topological measure of the protein structure network**	No	No	Yes
**Side chain information**	No	Yes (side chain pK_a)	Yes (side chain ASA)
**Feature extraction method**	Concatenated encoding of neighboring residues	Concatenated encoding of neighboring residues	Weighted average encoding of neighboring residues
**Dimensionality of encoding vectors**	315	253	43
**Threshold-dependent evaluation measure**	No	Yes (ROC-AUC)	Yes (PR-AUC)
**Significance of the difference between methods for comparison**	No	No	Yes

References

1. Tjong H, Zhou HX (2007) DISPLAR: an accurate method for predicting DNA-binding sites on protein surfaces. Nucleic Acids Res 35: 1465-1477.

2. Xiong Y, Liu J, Wei DQ (2011) An accurate feature-based method for identifying DNA-binding residues on protein surfaces. Proteins-Structure Function and Bioinformatics 79: 509-517.
